# Editorial: Mechanisms during bacterial infection: cellular recognition, signalling, and regulation

**DOI:** 10.3389/fcimb.2023.1286423

**Published:** 2023-09-26

**Authors:** Seung-Hak Cho, Cheorl-Ho Kim

**Affiliations:** ^1^Division of Zoonotic and Vector Borne Disease Research, Center for Infectious Disease Research, Korea National Institute of Health, Cheongju-si, Republic of Korea; ^2^Department of Biological Sciences, College of Science, Sungkyunkwan University, Suwon, Republic of Korea

**Keywords:** mechanisms of bacterial infection, cellular recognition, signaling, regulation, innate immunity, bacterial pathogen, host interaction, protein-protein interaction

This Research Topic entitled “*Mechanisms during Bacterial Infection: Cellular Recognition, Signaling, and Regulation*” aimed to explore the emerging effector molecules associated with pathogenic adhesion to host cells, the roles of effector molecules in pathways, and host cell responses to pathogen-mediated intracellular changes. The concepts and goals of the glycan-based molecules described in this topic may be new to our researchers; so far, four articles (two original articles and two review articles) have been successfully published.

Interactions between pathogenic bacteria and host cells, such as epithelial cells, trigger transmission of the initial upstream signals to cellular response machines. The transmitted information functionally induces related upstream signaling receptors and integrins expressed on cell surfaces. Continued signaling flows through downstream machinery to activate many intracellular kinases and transcription factors, and upregulate expression levels of target genes associated with pro-inflammatory cytokines. Consequently, the expression of epithelial surface markers is altered. Bacterial pathogens infections rely on the pathogen’s ability to evade the recognition and interaction pathways of the hosts- pathogens’ ability to evade host immune surveillance. The evasion leads to host immune dormancy, in which intracellular signaling and regulation associated with the host’s immune response are not activated.

Pathogens use multiple strategies to evade host immune responses. For example: 1) Certain pathogenic bacteria recruit platelets into the infectious process to acquire resistance to platelets’ antimicrobial properties (Kim, 2022a). Representatively, *Salmonella* bacteria have evolved to evade or subvert host immune surveillance. Conversely, the regulation of signaling pathways in host cells aids in pathogenic intrusion. 2) Pathogenic bacteria interact with the host surface molecules, such as glycans and proteins, to exploit the host’s intracellular signaling machinery, and consequently avoid host immune responses. At mechanistic and molecular levels, pathogenic bacteria interactions with hosts are based on specific recognition, binding, interaction, and adhesion, as well as the regulation of host cellular signaling.

Although a variety of cellular adhesion factors that are associated with interactions between pathogens and host cells in epithelial cells have been reported, information on the role of glycan in infections and signaling is limited. Most studies have focused on pathogenic factors-related symptoms in the pathogenesis of bacterial infections. Glycan-based pathogenicity, a lectin-glycan interaction (LGI) concept, has been proposed (Cho et al., 2022). LGIs can be systemically expanded during the immune response by the host glycans in the pathogenesis of infectious diseases (Kim, 2022a; Kim, 2022b). Notably, hosts and bacteria have evolved and use the same machinery, in which bacterial pathogens and hosts reciprocally utilize own proteins and glycans to invade and progress, respectively.

Yang et al. described the effect of coaggregation between two bacteria on human gingival epithelial cells (hGECs). The known subgingival plaque biofilms produced by *Fusobacterium nucleatum* were involved in the development of periodontitis. Another gingival pathogen, *Streptococcus gordonii*, coaggregated with periodontal pathogens and colonized the hGECs. Coaggregation between *F. nucleatum* and *S. gordonii* synergized subgingival plaque biofilms virulence. Although the coaggregation inhibited *F. nucleatum* adhesion and invasion into hGECs, it enhanced *S. gordonii* adhesion to hGECs. Mechanically, coaggregation increased the levels of innate immune receptors of Toll-like receptor (TLR)2 and TLR4 in hGECs. In addition, coaggregation inhibited hGEC apoptosis and promoted the secretion of proinflammatory cytokines tumor necrosis factor (TNF)-α and interleukin (IL)-6 by hGECs through TLR2/TLR4/NF-κB/MAPK signaling. However, coaggregation inhibited the secretion of anti-inflammatory cytokine transforming growth factor (TGF)-β1, to upregulate the p65/p38/JNK/NF-κB/MAPK signaling axis, and consequently, aggravated the host inflammatory response. Therefore, coaggregation suppressed the adhesion and invasion of *F. nucleatum* but promoted *S. gordonii* adhesion to hGECs.

Zhang et al. described *Fusobacterium necrophorum*, a pathogenic inducer of bovine foot rot characterized by a strong inflammatory response. Using a cow skin explant model, the authors reported that *F. necrophorum* bacillus induced inflammation with tissue cell apoptosis. *F. necrophorum* infection upregulated p-IκBα and nuclear factor (NF)-κB p65 levels via the NF-κB p65/TNF-α/IL-1β/IL-8 pathway, inducing an inflammatory response, and causing foot rot in dairy cows.

Singh et al. described membrane phospholipase-C (PLC) as a determinant of host-pathogen interaction in bacterial infections and bacterial infections pathogenesis in humans. Virulence factors are also associated with pathogenic bacteria survival, proliferation, and colonization in the host. Apart from protein interactions, the authors also identified PLC, an enzyme virulence factor, as a cell signaling and regulatory factor. PLC catalytically hydrolyzes membrane phospholipid to di-acyl-glycerol and inositol triphosphate. The products act as secondary messengers to activate signaling involved in the immune response. The involvement of PLCs in infectious diseases has not been well explained to date, although both the host and pathogen PLCs are directly linked to infections, pathogenesis, and disease symptoms. The authors systemically reviewed the pathogenic determinant PLC in infections, host recognition, and pathogenesis.

Jia et al. described *Porphyromonas gingivalis*-caused insulin resistance and its signaling. The *Porphyromonas gingivalis* (*P. gingivalis*), a gram-negative oral anaerobic bacteria, produced multiple toxins which contributed to periodontal pathogenesis and systemic diseases. The virulence factors disturbed the host’s innate and adaptive immunities to evade the host’s immune surveillance. In their study, for insulin resistance caused by *P. gingivalis*, bacteria-induced systemic inflammation disturbed insulin signaling through dysfunctional pancreatic β-cells and lowered insulin sensitivity-based insulin resistance. The authors presented a systematic overview and discussed the literature on *P. gingivalis*-driven insulin resistance and the relationship between insulin resistance as a systemic disease and *P. gingivalis* infection. Then, therapeutic approaches were proposed with insight into the current research directions and systemic disease-blocking.

In summary, the two original articles and two reviews present the latest information and most recent research results on bacterial and cellular recognition, signaling, and the regulation of innate immune aspects. Bacterial/host innate immunity is an immunological defense that involves binding on certain functional target molecules, such as glycans, proteins, and enzymes. The target molecules are directly associated with human diseases and also act against pathogenic invaders and self-associated patterns. This review provides an overview of basic innate immunity and the mechanisms of pathogenic bacteria and host cell interactions during infections and the inflammatory response. The articles that were reviewed were studies on pathogenic bacteria signaling and therapeutic approaches. This Topic provides insight into the emerging effector molecules associated with pathogenic adhesion to host cells and relevant treatment strategies for infectious diseases.

## Author contributions

S-HC: Writing – review & editing, Conceptualization. C-HK: Writing – original draft, Writing – review & editing, Conceptualization, Funding acquisition, Investigation, Methodology, Project administration.
